# New Drugs. Appliances, and Things Medical

**Published:** 1890-04-12

**Authors:** 


### NEW DRUGS, APPLIANCES, AND THINGS MEDICAL.

[All preparations, appliances, novelties, etc., of which a notice is desired, should be sent to The Editor, The Lodge, Porchester Square, W.]

### "THE AUTOMATIC RESPIRATOR."

Dr. C. R. Vanderburg, lecturer on pathology at Starling Medical College, Columbus, publishes "A device for forcing respiration, with a report of its use in three cases of opium narcosis" (New York *Medical Record*). The older methods, those of Sylvester, Hall, Howard, and others, depend upon the resiliency and elasticity of the chest-walls, lungs, and diaphragm. And Dr. Fell recommends tracheotomy, inserting a tube into the wound, and connecting this with bellows. The device the author suggests is called "The Automatic Forced Respirator." It consists of a heavy flexible rubber cup fitting over mouth, nose, and chin, and retained either by hand or straps about the head. In this cup is a brass tube, which connects with a bellows by a rubber tube about five feet long. Inside the brass tube are two valves, which are so constructed that they automatically control the inhaled and exhaled air, one directing the inhaled air from the bellows into the lungs, the other giving exit to exhaled air to the outside through the walls of the cup. The operator must work the bellows at the rate he wishes respiration to be forced, and the respirator does the rest. When this device is used air is forced into the lungs; it is then expired, as in natural respiration, as soon as pressure on the bellows is removed by the resiliency of the chest walls, lungs, and diaphragm, as in natural expiration. It resembles natural respiration in that expiration is a passive act. Three cases of poisoning by morphine are detailed, treated successfully by this instrument. There is, however, an objection to the use of bellows, as the lung tissue may be ruptured therefrom. But Dr. Vanderburg thinks this is a mistake. He states he has repeatedly endeavoured to rupture the lungs of a fowl by placing a blow-pipe in the trachea, with the chest laid open, giving the lungs no support, and he could not do so. And he instances the pressure a cornet-player uses to force a high note, without damage to the lungs. Again he says a given pressure in the trachea is manifoldly decreased in the air cells, owing to the expansion of the lung-tissue, and consequent distribution of pressure. Notwithstanding all this, we should imagine that injury to the lungs would depend on the amount of force used, and on the condition and strength of the lungs, which, of course, varies in different individuals. In the first case reported, forced respiration was conducted during three hours, in the second case over two hours, and in the third about two hours. Should further investigation accord with the author's experience, credit must be awarded to Dr. Vanderburg for a great improvement in the treatment of narcosis.

